# A Case of General Miliary Tuberculosis Involving the Choroid

**Published:** 1879-03

**Authors:** Edward L. Holmes

**Affiliations:** Chicago


### ARTICLE V.

A CASE OF GENERAL MILIARY TUBERCULOSIS INVOLVING THE CHOROID. By EDWARD L. HOLMES, M. D., Chicago. Read before the West Chicago Medical Society, Jan. 13th, 1879.

Through the kindness of Dr. W. T. Belfield I am permitted this evening to present to you this specimen. It is, I presume, the first specimen seen in the Northwest, which was removed from the cadaver at a post-mortem examination, for the purpose of verifying the clinical fact that the choroid is often implicated in acute miliary tuberculosis.

I believe Dr. C. Fenger, Pathologist of the Cook County Hospital, is the first physician who ever made such an autopsy in this portion of the country.

The literature upon the subject is found principally in foreign medical journals, especially in those devoted to ophthalmology.

In this special case I am informed that the patient, about thirty-five years of age, was brought to the County Hospital almost moribund, with no one to give the previous history. Miliary tubercles were found in nearly every organ of the body.

In this specimen eight minute white elevations are perceived in the posterior half of the choroid. The microscopic appearances are identical with those found in tubercle of other organs and are sufficiently well described in works on pathology.

The principal facts regarding this condition of the choroid are as follows:

1st. So far from being rarely found in the choroid, miliary tubercles find in this membrane quite a "favorite" locality for their development.

2d. They vary in size from one-third of a millimeter to two and one-half millimeters in diameter. As they are usually located in the posterior portion of the choroid, they can be recognized by means of the ophthalmoscope during life. As far as I know, however, the patients have all been on the verge of dissolution when the examinations were made.

3d. In no cases of extensive cheesy masses, confined to the lungs and abdominal organs, are tubercles found in the choroid.

4th. Whenever tubercular deposits are found in the choroid, they are of the miliary form and are present in " almost all the organs of the body," and especially in the meninges.

5th. So far as records state, no subjective symptoms have been observed. The patients have been either too young, or too near death to express complaints regarding disturbed vision. Theoretically elevations in the choroid, crowding upon the retina, especially near the macula, must cause distorted vision.

6th. Cohnheim and Graefe have shown that guinea pigs inoculated with tuberculous substance, died after a few weeks, tubercles being found in the choroid as well as in many other organs.

I may add that Manz and Graefe were perhaps the first who studied this subject. More recently tubercles have been found in the retina, optic nerve, ciliary bodies and iris in individual cases. As early as 1829, V. Ammon and others described as tubercle large masses of " cheesy" substance in the globe, which may have been produced by retrograde degeneration of malignant growths.

---

IN remembrance of the cordial reception and most generous hospitality he met with in the United States, and to meet the wishes of numerous friends, Dean Stanley has collected into a volume his various addresses and sermons, which Macmillan & Co. will publish in a few days; still further to increase the interest of the volume, the publishers, by the kind permission of the Dean, have been able to add a photograph portrait.

THE very unpleasant, pungent odor of iodoform can be completely masked by oil of peppermint. For instance, iodoform 2.0, vaseline 30.0, rubbed up with six drops of oil of peppermint, make an ointment with a pleasant aromatic scent.

THERE are seventeen teachers of otology at fourteen universities of the German empire, while at six universities otology has found no representation yet in the medical faculties.

